# Impact of polyphenols on heart failure and cardiac hypertrophy: clinical effects and molecular mechanisms

**DOI:** 10.3389/fcvm.2023.1174816

**Published:** 2023-05-24

**Authors:** Neda Hedayati, Alireza Yaghoobi, Marziyeh Salami, Yasaman Gholinezhad, Farnaz Aghadavood, Reza Eshraghi, Mohammad-Hossein Aarabi, Mina Homayoonfal, Zatollah Asemi, Hamed Mirzaei, Mohammad Hajijafari, Alireza Mafi, Malihe Rezaee

**Affiliations:** ^1^ School of Medicine, Iran University of Medical Science, Tehran, Iran; ^2^ Department of Pharmacology, School of Medicine, Shahid Beheshti University of Medical Sciences, Tehran, Iran; ^3^ Department of Clinical Biochemistry, School of Medicine, Shahid Sadoughi University of Medical Sciences, Yazd, Iran; ^4^ Department of Pharmacology, School of Medicine, Shahid Beheshti University of Medical Sciences, Tehran, Iran; ^5^ Student Research Committee, Isfahan University of Medical Sciences, Isfahan, Iran; ^6^ School of Medicine, Kashan University of Medical Sciences, Kashan, Iran; ^7^ Student Research Committee, Kashan University of Medical Sciences, Kashan, Iran; ^8^ Department of Clinical Biochemistry, School of Pharmacy and Pharmaceutical Sciences, Isfahan University of Medical Sciences, Isfahan, Iran; ^9^ Research Center for Biochemistry and Nutrition in Metabolic Diseases, Institute for Basic Sciences, Kashan University of Medical Sciences, Kashan, Iran; ^10^ Department of Anesthesiology, School of Medicine, Kashan University of Medical Sciences, Kashan, Iran; ^11^ Nutrition and Food Security Research Center, Isfahan University of Medical Sciences, Isfahan, Iran; ^12^ School of Medicine, Shahid Beheshti University of Medical Sciences, Tehran, Iran; ^13^ Tehran Heart Center, Cardiovascular Diseases Research Institute, Tehran University of Medical Sciences, Tehran, Iran

**Keywords:** polyphenols, oxidative stress, inflammation, heart failure, targeted therapy

## Abstract

Polyphenols are abundant in regular diets and possess antioxidant, anti-inflammatory, anti-cancer, neuroprotective, and cardioprotective effects. Regarding the inadequacy of the current treatments in preventing cardiac remodeling following cardiovascular diseases, attention has been focused on improving cardiac function with potential alternatives such as polyphenols. The following online databases were searched for relevant orginial published from 2000 to 2023: EMBASE, MEDLINE, and Web of Science databases. The search strategy aimed to assess the effects of polyphenols on heart failure and keywords were “heart failure” and “polyphenols” and “cardiac hypertrophy” and “molecular mechanisms”. Our results indicated polyphenols are repeatedly indicated to regulate various heart failure-related vital molecules and signaling pathways, such as inactivating fibrotic and hypertrophic factors, preventing mitochondrial dysfunction and free radical production, the underlying causes of apoptosis, and also improving lipid profile and cellular metabolism. In the current study, we aimed to review the most recent literature and investigations on the underlying mechanism of actions of different polyphenols subclasses in cardiac hypertrophy and heart failure to provide deep insight into novel mechanistic treatments and direct future studies in this context. Moreover, due to polyphenols' low bioavailability from conventional oral and intravenous administration routes, in this study, we have also investigated the currently accessible nano-drug delivery methods to optimize the treatment outcomes by providing sufficient drug delivery, targeted therapy, and less off-target effects, as desired by precision medicine standards.

## 1. Introduction

Natural compounds derived from plants, marine organisms, and animals have attracted considerable attention due to their benefits for the health of the human body. It has been shown that natural products have bioactive properties and can improve various chronic diseases at the molecular level (1). Polyphenols contain one or more phenolic rings and are abundant in red fruits, vegetables, nuts, cocoa, wine, grapes, and tea (2, 3). These natural compounds have received wide attention recently regarding the well-known therapeutic effects and potential applications of polyphenols (4, 5). Many studies have highlighted the benefits of polyphenols in the prevention of various diseases such as cardiovascular diseases (CVDs), neurodegenerative diseases, diabetes, and cancer, which are mainly due to their ability to reduce reactive oxygen and nitrogen species, activate antioxidant enzymes, attenuate oxidative stress and inflammatory response (6–8).

CVDs describe a range of pathological conditions that affect the structure and function of the heart and blood vessels, which are responsible for the highest mortality worldwide, with an estimated 17.9 million deaths annually (9). Most CVDs could eventually lead to heart failure (HF), a clinical syndrome in which the heart cannot pump enough blood to meet the body's metabolic needs (10). HF is a critical public health problem with noticeable morbidity and mortality (11). In several conditions, the onset of HF is accompanied by cardiac hypertrophy (CH), defined as an adaptive reaction in response to chronic cardiac stress, such as hypertension, myocardial infarction (MI), valvular heart disease, as well as hereditary diseases that cause enlargement of cardiomyocytes and thickening of cardiac muscle fibers. At first, such growth maintains the function of the heart. However, chronic and persistent stress conditions lead to dilation of the ventricles, reduced contractile function, and eventual progression to HF (10, 12). Although drug therapies have partly successfully reduced HF mortality, most patients experience a downhill phase; therefore, more therapeutic measures are necessary (13).

Currently, pharmacological products derived from natural compounds, possessing unique structural and functional diversity, are regarded as promising adjutant approaches to prevent and treat CVD and HF (14, 15). Polyphenols are one of the natural compounds that can treat and prevent the progression of HF and CVD due to their various therapeutic properties, including anti-inflammatory, antioxidant, antiapoptotic, antiatherogenic, and antihypertension effects. Clinical and animal studies have reported that polyphenols reduce reactive oxygen species (ROS) and malondialdehyde (MDA) in the heart (16) and induce the expression of enzymes involved in the detoxification system, such as superoxide dismutase (SOD), catalase (CAT), and glutathione (GSH) peroxidase (17). In addition, polyphenols are indicated to inhibit the oxidation of low-density lipoproteins (LDL) and reduce the cytotoxicity caused by oxidized LDL (ox-LDL) in endothelial cells; therefore, polyphenols could prevent the onset and progression of atherosclerosis (18). On the other hand, studies conducted on both ischemic and non-ischemic HF animal models have demonstrated that resveratrol (RES), a principal polyphenol compound, improved systolic/diastolic ventricular function (19, 20), cardiac hemodynamic (21), and survival, as well as reduced left ventricular remodeling (22); all of which effects reduce the risk of HF.

Without a doubt, clearing the role of polyphenols will enhance knowledge of HF and CH and provide novel opportunities to extend more effective therapeutic approaches. Therefore, in this study, we aimed to summarize the findings regarding the potential positive effects of polyphenol compounds and signaling pathways and target molecules affected by several of these unique structures in HF and CH. We also highlighted the clinical roles of polyphenols that may provide promising therapeutic strategies in these pathological settings.

## 2. Pathophysiology of cardiac hypertrophy and heart failure

HF occurs under different pathological circumstances, including MI (23), valvular inadequacy (24), hypertension (25), cardiomyopathy (26), diabetes (27), and autoimmune diseases (28). Heart loss of function is primarily due to abnormal remodeling after the defects in cardiomyocytes' cellular metabolisms (29–31). Following hemodynamic alterations, compensatory mechanisms help maintain the heart's function, like the renin-angiotensin process, the sympathetic nervous system, and other neurohormonal mechanisms (32). Elevating the vasoconstrictors and neural hormones increases the cardiac muscle mass and empowers contractility, resulting in cardiac hypertrophy (33).

Physiological hypertrophy enhances heart performance by adding sarcomere units in parallel, which leads to a higher contractility; however, if the stress continues chronically, especially in the left ventricle, it eventually leads to pathologic hypertrophy and HF (12). Cardiac hypertrophy (CH) is the result of cardiomyocytes’ metabolism and calcium handling alterations, cardiac inflammation, cell death (e.g., apoptosis and autophagy), extracellular matrix (ECM) deposition, fibrosis, and angiogenesis (34). Sub-cellular changes in the heart, including oxidative stress, the increased entrance of Ca^2+^ in cells, and stimulant of proteases, combined with inflammation, lead to cardiac remodeling, pathological hypertrophy, and eventually, HF (35).

The metabolic changes in pathological hypertrophy led to using glucose instead of fatty acid oxidation to produce ATP as the primary energy source (36). In hypertrophic conditions, using less oxygen to produce ATP is more desirable (37). As the energy demand exceeds the supply, myocardial ATP levels decrease, and contractility becomes compromised, leading to HF development (33). Maintaining balanced ATP levels and Ca^2+^ concentration is imperative for healthy cardiac contractility. Intracellular levels of calcium increase in favor of improving contractile function during hypertrophy.

On the other hand, under metabolic alterations, sarco/endoplasmic reticulum Ca^2+^-ATPase (SERCA2a), an organelle that pumps calcium back into the sarcoplasmic reticulum, does not work correctly, which leads to the sizeable intracellular concentration of Ca^2+^ (38). Overload of Ca^2+^ enrolls pathways such as proteolytic enzymes, which promote HF (33, 35). The steady high amount of vasoactive hormones leads to the dominance of oxyradicals against antioxidants, boost redox-sensitive signals, and results in oxidative stress (39) and the formation of inflammatory cytokines, such as TNF-α, IL-6, and IL-1 (40). Besides, the inflammatory response activates macrophages and T immune cells (CD^3+^, CD^8+^, CD^4+^), which provoke abnormal heart remodeling and dysfunction of the left ventricle (41).

Fibroblasts are associated with maintaining cardiac contraction and signal pathways through forming the ECM. These cells develop ECM by secretion of humoral mediators, produce collagen type I and III, fibronectin, matrix metalloproteinases (MMPs), and tissue inhibitor of matrix metalloproteinases (TIMPs) (42). In the process of cardiac remodeling by inflammation, fibroblasts change into myofibroblasts. Myofibroblasts' constant activation and proliferation lead to the abnormal deposition (interstitial/replacement) and following accumulation of collagen in the heart that cause defective electrical conduction and inability to contractile function, which leads to cardiac dysfunction and HF (34, 42, 43). Loss of cardiomyocytes is a consequence of former apoptosis, fibrosis, and necrosis mechanisms (44, 45).

## 3. Polyphenols

Polyphenols are secondary metabolites in plants with more than one phenolic group and are synthesized from pentose phosphate, phenylpropanoid, and shikimate pathways (4). Based on the number of rings and the elements that connect these rings, phenolic compounds are divided into subclasses, including flavonoids, phenolic acids, stilbenes, and lignans (46). Flavonoids are the most common phenolic metabolites found abundantly in vegetables and fruits (47). They have a typical basic structure consisting of two aromatic rings, A and B, connected by an oxygen-containing heterocyclic ring, the C ring. Based on their C ring structure, flavonoid compounds can be divided into six categories: flavanols, isoflavones, flavones, flavanones, and anthocyanins, each of which has a different hydroxylation and methylation pattern of A and B rings (48). Quercetin, myricetin, hesperetin, naringenin, nobiletin, eriodicytol, and catechins are the most common flavonoids in the human diet (49). Flavonoids possess antioxidant activities owning to having several phenolic hydroxyl groups that neutralize active hydrogen atoms and prevent the auto-oxidation of lipids (50). In addition, many flavonoids exhibit various medicinal effects other than antioxidative effects. For instance, pinocembrin, quercetin, rutin, and catechin have anti-inflammatory, anti-tumor, anti-apoptotic, anti-microbial, anti-hyperlipidaemic, and anti-diabetic properties that make them suitable therapeutic candidates in various pathological conditions (51–55).

Phenolic acids are another critical group of polyphenols, constituting approximately one-third of the polyphenolic compounds in the human diet. This group is found in all plant materials, especially in fruits with an acidic taste (7). Hydroxybenzoic acid and hydroxycinnamic acid derivatives are the two major groups of phenolic acids. Hydroxycinnamic acids are characterized by a saturated tail containing a carboxyl group (C6H5CHCHCOOH) and include compounds such as ρ-hydroxycinnamic, ρ-coumaric, caffeic, and ferulic acids. The compounds derived from hydroxybenzoic acid only have carboxyl groups (COOH), and the most common ones are ρ-hydroxybenzoic, gallic, protocatechuic, and vanillic acids (53). Like structural diversity, the biological potential of phenolic acids has a broad spectrum, such as anti-inflammatory, antioxidative stress, anti-hyperglycemic, neutralizing free radicals, and anti-cancer effects (56).

Lignans are phenolic compounds consisting of two phenylpropane units (57). Secoisolariciresinol, matairesinol and lariciresinol are common lignans. These compounds are abundant in flaxseed; however, grains, nuts, and vegetables also contain small amounts of lignans (58). In the human body, lignans are metabolized by intestinal microflora into enterodiol and enterolactone, which have estrogen-like structures; therefore, lignans are being investigated for possible use in ameliorating cancer, especially breast cancer (59). In addition, many studies have shown that consuming foods rich in lignan has favorable effects in preventing and improving diseases such as hypertension, type 2 diabetes, metabolic syndrome, and CVDs (60–63). The last class of primary polyphenols is stilbenes, which consist of two phenyl groups connected by a two-carbon methylene bridge (64). One of the widely studied stilbene polyphenols is RES, which is abundant in the skin of red grapes; however, it is also present in lower concentrations in blueberries, raspberries, mulberries, and peanuts (65). RES has many biological properties, including antioxidant, anti-inflammatory, cardioprotective, neuroprotective, and anti-tumoral activities (66).

## 4. Polyphenols: a valuable strategy in the treatment of heart failure and cardiac hypertrophy

**Figure 1:**
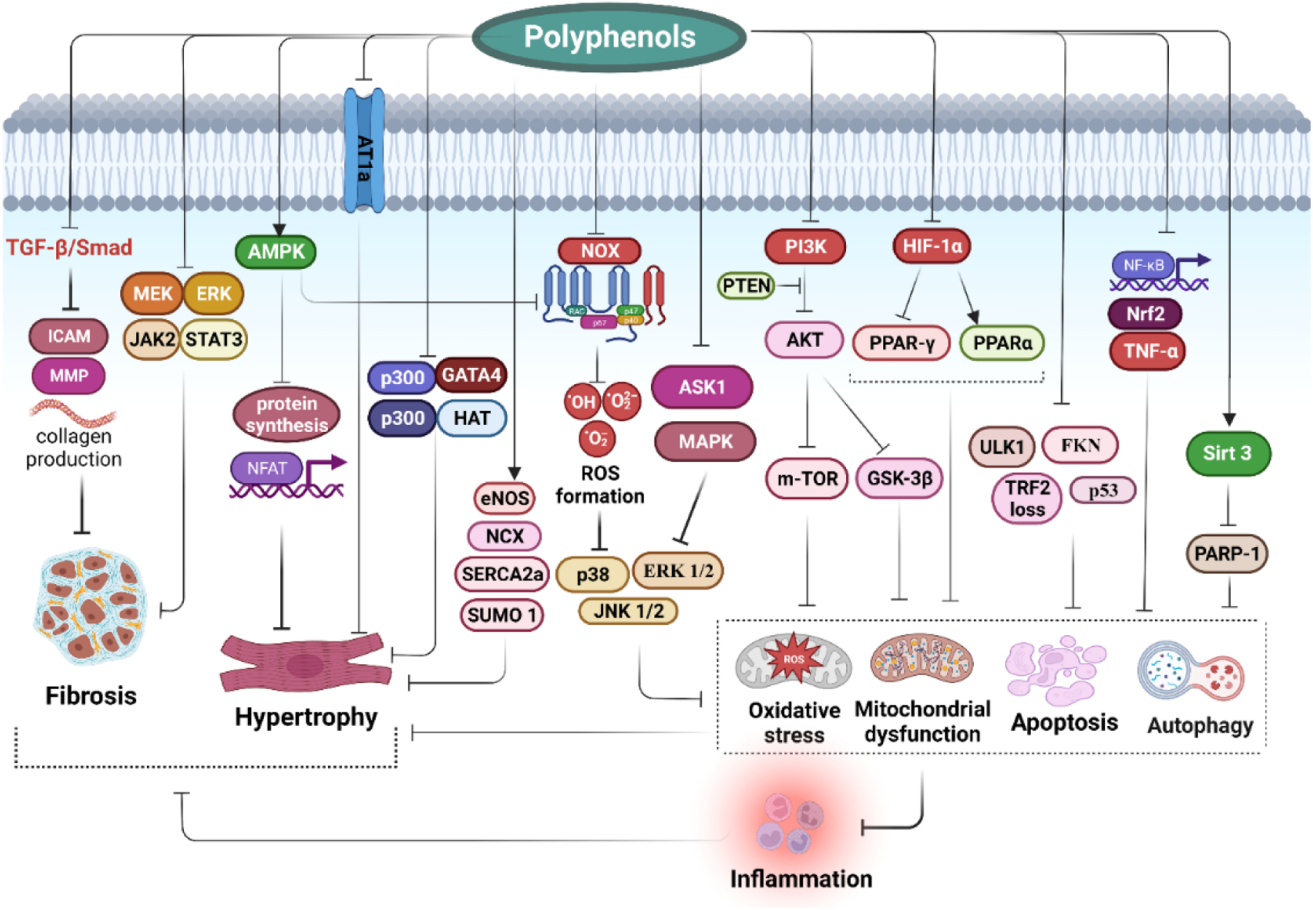
Impact of polyphenols on modulation of heart failure and cardiac hypertrophy: clinical effects and molecular mechanisms.

Polyphenols have many potential effects that can modify the involved processes in HF and cardiac CH via multiple mechanisms. Figure 1 represents several familiar and vital underlying mechanisms of polyphenols in preventing and treating HF. Excessive cardiac oxidative stress caused by upregulated nicotinamide adenine dinucleotide phosphate (NADPH)-oxidases (Nox) and increased ROS production in mitochondria are the main inducers of HF (67, 68). In addition, in conditions of severe oxidative stress, the antioxidant system is overwhelmed due to the inhibition of nuclear factor erythroid 2-related factor 2 (Nrf2) activity by nuclear factor kappa-light-chain-enhancer of activated B cells (NF-κB). Nrf2 and NF-κB are crucial pathways modulating the adequate balance of the status of cellular redox and react to inflammation and stress. The interplay between such signaling molecules controls via various elaborate molecular interactions and mostly is determined by the type of cells and tissues. These interactions act through transcriptional and post-transcriptional machinery leading to the adjustment of dynamic reactions to ever-altering environmental signals (69). Pharmacological and genetic investigations have confirmed the functional interplay between Nrf2 and NF-κB. The lack of Nrf2 aggravates NF-κB activity resulting in augmented cytokine production, whiles NF-κB regulates the transcription and activity of Nrf2, owning both positive and negative impacts on the expression of target genes. Both Nrf2 and NF-κB are modulated by elements sensitive to redox. The deficiency of Nrf2 is correlated with incremented oxidative and nitrosative stress resulting in augmentation of cytokine release since NF-κB is easily activated in oxidative conditions (70). Cells mostly counteract oxidative stress by activating antioxidant enzymes (such as catalase, glutathione peroxidase, and superoxide dismutase) and antioxidant compounds. The antioxidant response element (ARE) is the DNA sequence accountable for regulating the antioxidative and cytoprotective responses of the cells, while Nrf2 is the main modulator of the xenobiotic-activated receptor (XAR) responsible for triggering the ARE pathway. In this regard, the Nrf2-ARE signaling is a key cytoprotective system to protect cells against oxidative stress and provides an acceptable redox balance in cells (69). NF-κB is a redox-modulated transcription factor involved in the regulation of cell growth, apoptosis, and inflammation. Oxidative stress triggers the activation of IκB kinase (IKK) mediating the phosphorylation of IκB which finally elevates NF-κB release. NF-κB stimulates the transcription of proinflammatory agents such as cytokines, cyclooxygenase-2, iNOS, and vascular adhesion molecules. Nrf2 restricts the activation of NF-κB signaling via neutralizing ROS by antioxidants, therefore inhibiting activation of NF-κB via ROS mediation (71, 72). As a result, NF-κB is the main molecular target to improve the antioxidant system (70). It has been shown that some polyphenols prevent the degradation of IκB, the major NF-κB inhibitor protein, by inhibiting the phosphorylation or ubiquitination of IκB kinase, thus blocking the entry of the NF-kB p65 subunit into the nucleus. In addition, polyphenols interfere with the binding of NF-κB to DNA (73, 74).

Sirtuins are a group of NAD^+^-dependent deacetylases located in mitochondria, nucleus, and cytoplasm, with seven subgroups. Among them, sirtuin1 (SIRT1) inhibits NF-κB-induced inflammatory signaling (75). SIRT1, a redox-sensitive enzyme, is abundantly expressed in embryo rat hearts, and suppressing its expression leads to developmental disorders in the heart (76). Sirt1 is upregulated by polyphenols, such as RES, which was also found to induce the deacetylation of NF-κB in Sirt1 dependent manner, suppressing NF-κB activity (77). On the other hand, a study reported that gallic acid could attenuate oxidative stress by directly reducing the expression of Noxs such as Nox1, Nox2, and Nox4 in the cardiac tissue of hypertensive rat models (78).

In cardiomyocytes, mitochondria constitute approximately one-third of the cell volume, reflecting the high energy requirements of these cells (79). Mitochondrial dysfunction is another cause of HF and is characterized by excessive leakage of ROS that results from complexes I and III of the electron transport chain (ETC). This process leads to membrane depolarization and reduced ATP synthesis, followed by the release of cytochrome c (Cyt c) from mitochondria, ultimately triggering apoptosis (80). Anthocyanins, a subclass of flavonoids, have a high antioxidant capacity. A study showed that they act as Cyt c reductase and thus prevent the activation of caspases and inhibit apoptosis (81). Further investigations revealed that anthocyanins could act as electron acceptors in complex I of the mitochondrial respiratory chain, leading to increased ATP production after ischemia in rats (82).

Atherosclerosis is a chronic inflammatory condition characterized by the deposition of extracellular lipids and, eventually, the narrowing of the arteries (83). Atherosclerosis is also a significant cause of CVDs, such as coronary artery disease, the most common etiology of HF (84). Improving the lipid profile can be beneficial by considering the role of lipids, especially LDL, in the atherosclerotic process. The effectiveness of polyphenols on the lipid profile has been widely investigated. Many of these studies have shown that polyphenols could improve lipid disorders by reducing plasma levels of triglycerides and LDL-cholesterol and increasing high-density lipoprotein (HDL)-cholesterol (85, 86). In addition, it has been reported that RES increased the expression of LDL receptors on hepatocytes *in vitro*, thus helping to reduce blood LDL cholesterol levels further (87). On the other hand, it has been shown that polyphenols reduced ox-LDL by inducing the expression of the antioxidant system (88).

Red wine polyphenols improve hypertension, a leading cause of cardiac hypertrophy, by increasing the activity of nitric oxide synthase (NOS) and reducing end-organ damage such as myocardial fibrosis (89). Moreover, several preclinical and clinical studies have shown the antihypertensive properties of RES (90–92). Cumulatively, polyphenols present a potentially effective treatment for patients with HF and CH using a plant-based diet.

## 5. Polyphenols in HF: mechanistic aspects and therapeutic strategy

### 5.1. Resveratrol

Resveratrol (3, 5, 4′-trihydroxystilbene) is a phytoalexin with a polyphenol structure, and various plants naturally produce it as a defense mechanism against stress, such as infection and UV radiation. Grapes, peanuts, and peanut sprouts are considered primary dietary sources of RES (93–95). Several studies have reported that RES exerts multiple functions in the biological processes, such as anti-inflammatory, antioxidant, anticancer, neuroprotective, and cardioprotective (96, 97). The safety of RES has been evaluated in healthy individuals and it has been disclosed to regulate different indicators of various diseases. It has been reported in clinical trials that RES is safe up to 5 g/day and known as a safe phytochemical (98) According to its properties, RES has various beneficial effects on cardiovascular disorders, such as improving cardiac remodeling, function, fibrosis, and regulating cardiac metabolism and blood lipid (19, 99). A low incidence of CVDs in French people, despite their high-risk diet, could be associated with long-term red wine intake, which contains RES, and this is called the “French paradox” (100) (Table 1).

**Table 1:** Various mechanisms of polyphenols in improving the heart failure.

Treatment (nutraceuticals)	Dosage	Route of administration	During of administration	Subjects	Targeted signaling pathways	Effects	Ref.
Resveratrol	10 mg/kg/day	Oral gavage	4 weeks	Aldosterone-induced HF	TGF-β/Smad3/Sirtuin1	Decreases cardiac fibrosis	(101)
	450 mg/kg/day	Oral (in diet)	2 weeks	Pressure overload-induced HF	IRS-1, Akt, and AMPKα	Increases exercise capacity Enhanced skeletal muscle metabolism	(102)
	20 mg/kg/day	Intraperitoneal injection	42 days	MI-induced HF	FKN	Improves cardiac fibrosis Increases autophagy	(103)
	320 mg/kg/day	Oral (in diet)	2 weeks	Pressure overload-induced HF	AMPK	Improves cardiac metabolism and energy	(19)
	2.5 mg/kg/day	Oral	16 weeks	MI-induced HF	AMPK/SIRT1	Improves cardiac function and metabolism	(104)
	18 mg/kg/day	Oral (in diet)	8 weeks	Hypertension-induced HF	AMPK	Improves cardiac metabolism balance	(21)
	50, 100 µM	–	–	Phenylephrine-induced neonatal rat cardiomyocyte	LKB1/AMPK, Akt, p70S6K, eEF2, and NFAT/calcineurin signaling pathways	Suppress cardiac hypertrophy	(105)
	0.2 g/kg/day	Oral (in diet)	2 weeks	MI-induced HF	CYP1B1 and cardiotoxic HETE metabolites	Improves cardiomyocyte metabolism	(22)
	80 mg/kg/day	Intragastric	4 weeks	ISO-induced cardiac hypertrophy	Cyt c, Bcl-2, and Atg5	Decrease apoptosis Increase autophagy Inhibit cardiac hypertrophy	(106)
	10 mg/kg/day (+ Atorvastatin 10 mg/kg/day)	Oral	25 days	ISO-induced cardiac hypertrophy	–	Reduces cardiac hypertrophy	(107)
	2.5 mg/kg/day	Oral gavage	28 days	Pressure-overload cardiac hypertrophy	–	Reduces oxidative stress Suppresses development and progression of cardiac hypertrophy	(108)
	25 and 50 mg/kg/day	Oral gavage	2 weeks	TCA-induced cardiac hypertrophy	PTEN, AKT/mTOR, AMPK Immunoproteasome	Decreases cardiomyocyte hypertrophy	(109)
	30 mg/kg/day	Oral gavage	5 weeks	CIH-induced cardiac hypertrophy	PI3K/AKT/mTOR	Increases autophagy Inhibits apoptosis Reduces oxidative stress	(110)
	150 mg/kg/day	Oral gavage	4 weeks	Pressure overload-induced cardiac hypertrophy	miR-155, BRCA1, FoxO3a	Improves cardiomyocyte hypertrophy	(111)
	320 mg/kg/day	Oral (in diet)	5 weeks	Hypertension-induced cardiac hypertrophy	LKB1/AMPK/eNOS, p70S6k	Prevents cardiomyocyte hypertrophy	(90)
	1 mg/kg/day	Intraperitoneal	35 days	Abdominal aortic banding-induced cardiac hypertrophy	Ang II and AT1a receptor	Suppresses cardiomyocyte hypertrophy	(112)
	2.5 mg/kg/day	Oral gavage	10 weeks	Hypertension-induced cardiac hypertrophy	–	Decreases oxidative stress Inhibits cardiomyocyte hypertrophy	(113)
	10 or 50 mg/kg/day	Oral gavage	4 weeks	Hypertension-induced cardiac hypertrophy	NO, ET-1 and AngII	Improves cardiomyocyte hypertrophy	(91)
Curcumin	100 mg/kg/day	Oral	8 weeks	Pressure overload-induced HF	MMP-2 and MMP-9	Reduces collagen deposition Improves cardiac remodeling	(114)
	100 mg/day/kg	Oral	10 weeks	Volume and pressure overload-induced HF	DDK-3/p38/JNK/ ASK1	Decreases HF markers Improves cardiac function	(115)
	50 mg/kg/day	Gastric gavage	7 weeks	PE-induced cardiac hypertrophy	p300/GATA4	Represses hypertrophic responses in cardiomyocytes	(116)
	200 mg/kg/day	Intragastric	4 weeks	ISO-induced cardiac hypertrophy	mTOR	Attenuates cardiac hypertrophy and fibrosis Increases autophagy	(117)
	100 µg/kg	Intraperitoneal	12 or 24 h	Lipopolysaccharide-induced cardiac hypertrophy	p300-HAT	suppresses cardiomyocyte hypertrophy	(118)
	50 mg/kg/day	Gastric gavage	9 weeks	TCA-induced cardiac hypertrophy	Na^+^/Ca2^+^ exchanger (NCX) and eNOS	Decreases cardiomyocyte hypertrophy	(119)
Quercetin	20 mg/kg/day	Intragastric	8–12 weeks	Hypertension-induced cardiac hypertrophy	PARP-1/SIRT3	Improves mitochondrial dysfunction	(120)
	10 mg/kg/day	Oral	4 days	ISO-induced cardiac hypertrophy	–	Exerts anti-oxidant effects Improves mitochondrial dysfunction Decreases oxidative stress	(121)
	5, 10, or 20 mg/kg/day	Intragastric	8 weeks	AAC-induced cardiac hypertrophy	Proteasome/GSK-3α/β AKT and LKB1/AMPKα	Decreases cardiomyocyte hypertrophy Reduces excessive collagen deposition	(122)
	25 mM/kg/day	Intraperitoneal	2 days	Ang II-induced HF	–	Reduces cardiac fibrosis, inflammation and myocardial hypertrophy	(123)
	5 mg or 10 mg/head/day	Oral	3 weeks	AAC-induced cardiac hypertrophy	ERK1/2, p38 MAP kinase, Akt and GSK-3beta	Reduces oxidant status Attenuates cardiomyocyte hypertrophy	(124)
	1.5 g/kg	Oral	7 days	AAC-induced cardiac hypertrophy	ERK1/2	Reduces cardiomyocyte hypertrophy	(125)
Gallic acid	5 or 20 mg/kg	Oral gavage	8 weeks	TAC-induced cardiac hypertrophy	JAK2, STAT3, ERK1/2, AKT, and NFATc1 ULK1	Decreases cardiac myocyte hypertrophy, fibrosis, inflammation, and oxidative stress activates autophagy	(126)
	100 mg/kg/day	Oral	2 weeks	TAC-induced cardiac hypertrophy	TGF-β1/Smad	Reduces cardiac fibrosis	(127)
	100 mg/kg/day	Oral	2 weeks	TAC-induced HF	Epithelial-mesenchymal transition (EMT)-related genes and factor	Reduces cardiac fibrosis	(128)
	320 mg/day	Oral	16 weeks	Hypertension-induced cardiac hypertrophy	Nox2, GATA4	Attenuates cardiomyocyte hypertrophy Reduces oxidative stress	(78)
	100 mg/kg/day	Intraperitoneal	3 weeks	ISO-induced cardiac hypertrophy	JNK2, Smad3	Attenuates cardiomyocyte hypertrophy Reduces cardiac fibrosis	(129)
Genistein	1 mg/kg/day	Subcutaneous	10 days	PH-induced RV hypertrophy and failure	ER-β receptor	Decreases cardiomyocyte hypertrophy	(130)
	40 mg/kg/day	Oral	7 weeks	Pressure overload-induced cardiac hypertrophy	MAPK and AKT/GSK-3β signaling pathways	Reduces cardiomyocyte hypertrophy	(131)
	0.1 and 0.2 mg/kg/day	Subcutaneous	14 days	ISO-induced cardiac hypertrophy	NOS enzymes (iNOS and eNOS)	Attenuates cardiomyocyte hypertrophy Reduces cardiac fibrosis Reduces oxidative stress	(132)
	100 mg/kg	Oral (in diet)	21 days	ISO-induced cardiac hypertrophy	miR-451/TIMP2	Attenuates cardiomyocyte hypertrophy	(133)
Pterostilbene (complexed with hydroxypropyl-β-cyclodextrin)	25, 50, or 100 mg/kg/day	Oral	2 weeks	PAH-induced RV failure	–	Improves cardiomyocyte metabolism Attenuates oxidative stress	(134)
Kaempferol	20 mg/kg/day	Oral	42 days	Diabetic-induced HF	Nrf2, NF-κβ, and PI3K/Akt/GSK-3β signaling pathways	Exerts anti-oxidant, anti-inflammatory and anti-apoptotic effects	(135)
Luteolin	10 ug/Kg/day	Oral	14 days	Ischemia/reperfusion-induced HF	PI3K/Akt pathway, SUMO1 pathway, SERCA2a, PLB	Improves cardiomyocyte contractibility	(136)
Apigenin	20 μg/h	Infusion	4 weeks	Hypertension-induced cardiac hypertrophy	NADPH oxidase (NOX)	Decreases ROS generation Decreases inflammation	(137)
	50–100 mg/kg	Oral gavage	4 weeks	Hypertension-induced cardiac hypertrophy	HIF-1α PPARα, CPT-1 and PDK-4 PPARγ, GPAT, and GLUT-4	Improves cardiomyocyte metabolism Decreases cardiomyocyte hypertrophy	(138)
Epigallocatechin gallate	200 mg/kg/day	Oral	30 days	Aging-induced HF	TGFβ/TNFα/NF-κB	Prevents cardiac hypertrophy Decreases cardiac fibrosis and apoptosis	(139)
	50, 100 mg/kg/day	Intragastrical	6 weeks	Pressure-overload cardiac hypertrophy	TRF2	Reduces oxidative stress and apoptosis	(140)
Kaempferol	100 mg/kg/day	Oral	6 weeks	Aorta banding-induced cardiac hypertrophy	ASK1/MAPK and ASK1/JNK1/2/p38	Reduces oxidative stress Suppresses apoptosis	(141)
Delphinidin	15 mg/kg/day	Intraperitoneal	8 weeks	TAC-induced cardiac hypertrophy	AMPK/NOX/MAPK ERK1/2, P38 and JNK1/2	Decreases oxidative stress Inhibits cardiomyocyte hypertrophy	(142)
Caffeic acid	100 mg/kg/day	Oral gavage	6 weeks	Aorta banding-induced cardiac hypertrophy	MEK/ERK TGF-β/Smad	Reduces cardiomyocyte hypertrophy and fibrosis	(143)
Hesperidin	200 mg/kg/day	Oral	28 days	ISO-induced cardiac hypertrophy	PPAR-γ	Exerts anti-inflammatory, anti-apoptotic, and anti-oxidant effects	(144)

#### 5.1.1. Resveratrol regulates cardiac metabolism

The dysregulated metabolic state contributes significantly to the progression of HF, leading to metabolism disorders. AMP-activated protein kinase (AMPK) is an energy regulator that controls cells’ metabolic function and balances ATP generation and consumption. AMPK is activated by an increased cellular AMP-to-ATP ratio, shifting cell metabolism towards the ATP-generation pathway to restore cellular ATP levels (145, 146). Previous studies have shown that AMPK activation can decelerate HF progression (147). Some *in vitro* and *in vivo* studies have shown that Sirt1 exerts antioxidative effects by activating the AMPK pathway. GU et al. found that RES (2.5 mg/kg/day, 16 weeks) benefits HF mice's survival rate by upregulating AMPK through Sirt1 activation (104). Low-dose treatment with RES was demonstrated to alleviate cardiac remodeling in MI-induced HF rats. Matsumura et al. showed that RES (0.2 g/kg/day, two weeks) restored levels of fatty acid oxidation and enhanced cardiac energy metabolism in Male Sprague-Dawley (SD) rats. Inhibition of the activity of cytochrome P450 1B1 (CYP1B1), resulting in decreased expression of its metabolites, especially mid-chain Hydroxyeicosatetraenoic acid (HETE) metabolites, has been suggested as a possible pathway of cardioprotective effects of RES (22). HETEs is a cardiotoxic metabolite of arachidonic acid (AA) catalyzed by CYP1B1, which induces cardiac fibrosis, systolic dysfunction, and vasoconstriction (148, 149). Additionally, RES suppressed the development and progression of cardiac remodeling and dysfunction in pressure-overload (PO)-induced hypertrophy. RES treatment (2.5 mg/kg/day, 28 days) alleviated lipid peroxidation (LPO) in Male Sprague-Dawley rats, indicating that improving effects of RES on CH is associated with its direct antioxidant effect on the heart (108).

In a study by Rimbaud et al., RES treatment (18 mg/kg/day, 8 weeks) in hypertensive Dahl salt-sensitive rat models was associated with preventing cardiac hypertrophy, remodeling, and dysfunction. RES Improved the cholesterol profile and prevented reduction in Mfn1 and OPA1 expression (necessary for mitochondrial fusion), cardiomyocyte oxidative capacity, and citrate synthase. In addition, RES protects the heart tissue against impaired fatty acid oxidation (FAO), which is dysregulated in HF. In contrast to untreated hypertensive models, RES-treated rats showed no decreased expression of PPARα (a gene that regulates FAO), CPT-1b (a gene involved in fatty acid transport), and utilization of carbohydrates (PDK4) (21). Similarly, the results of Sung et al. determined that RES (320 mg/kg/day, 2 weeks) could exert its effects on myocardial metabolism in C57Bl/6 mice by enhancing antioxidant defense enzymes, shifting heart metabolism from FAO towards glucose oxidation by activating AMPK, and increasing insulin sensitivity. Besides, collagen density, transcription of genes participating in cardiac fibrosis, and expression of MMP2 were reduced after treatment with RES, suppressing cardiac fibrosis and improving diastolic dysfunction (19). Moreover, the benefits of RES on the improvement of diastolic function and cardiac energy have been shown in mice metabolism with transverse aortic constriction (TAC)-induced HF. Treatment with RES decreased the expression of gene-related CH, such as *anf*, *bnp*, *β-mhc*, and *ska*, to the near-normal level, suggesting its positive effects on cardiac remodeling and HF on the transcriptional level (19).

#### 5.1.2. Resveratrol attenuates cell death, oxidative stress, and inflammation

Inflammatory processes mediated by cytokines promote the development and progression of HF (150). Previous studies revealed that CX3CL1, also known as fractalkine (FKN), is a transmembrane cytokine primarily expressed in endothelial and epithelial cells, and its expression is induced by stress. FKN is one of the most overexpressed cytokines in HF (151). RES has been shown to regulate the expression of FKN and suppress FKN-mediated inflammatory response (152). In this regard, Xuan et al. revealed that FKN is associated with MI- or PO-induced cardiac remodeling and HF. Based on their results, injecting RES (20 mg/kg/day, 42 days) into mice's hearts with Transverse aortic constriction (TAC) was correlated with acute phase amelioration in MI-induced HF and improved survival rate. Expression of atrial natriuretic peptide (ANP), intercellular adhesion molecule 1 (ICAM-1), and MMP-9, which participate in cardiac fibrosis, was increased in HF mice, and RES treatment reversed these changes. Further investigation showed that RES (20 mg/kg/day, 42 days) suppresses FKN and FKN-mediated inflammation to improve HF in mice models (103, 153). Aligned with previous studies, transfecting FKN into cardiomyocytes, fibroblasts, and endothelial cells, led to increased expression of ANP, ICAM-1, MMP-9, TGF-β, procollagens I and III in cells *in vitro* while treating with RES reversed all these results. Exposing neonatal rat cardiomyocytes to RES led to increased autophagosomes and overexpression of LC3-II and Atg5 proteins (participate in autophagy) while treating these cells with FKN reversed the effects of RES, indicating contrary effects of FKN on HF. This study suggests that RES collectively improves HF conditions and complications by antagonizing the effects of FKT (103).

Moreover, RES treatment (10 mg/kg/day, 4 weeks) in the murine model of heart failure decreased ROS levels and increased CAT, SOD, and GSH, indicating the role of RES in reducing oxidative stress. Moreover, the expression of collagen-I and -III and TGF-β (cardiac fibrosis factors) decreased after RES treatment, and the expression of phosphorylated eNOS (a marker for cardiac stiffness) was increased after treatment with RES, indicating that RES reduces cardiac fibrosis and stiffness (101). Silent information regulator 1 (SIRT1) is a class III histone deacetylase that protects the cardiovascular system against oxidative stress by boosting antioxidant defense and inhibiting apoptosis, inflammation, and autophagy (154–156). RES is an activator of SIRT1, which exerts cardioprotective effects by enhancing its expression. It has been indicated that RES reduces CH and cardiac fibrosis by activating SIRT1, leading to suppression of the TGF-β1/p-Smad3 signaling pathway (157). The TGF-β/Smad3 signaling pathway is an essential regulator of fibrosis in various organs, including the heart (158, 159). This study suggested inhibition of the TGF-β/Smad3 signaling pathway as the RES mechanism in suppressing cardiac fibrosis (101). Sirt1 is a primary regulator of cell metabolism, and recent studies revealed its role in oxidative stress and fibrosis (160, 161). Previous studies have shown that increased activity of Sirt1 reduces cardiac fibrosis by negatively targeting Smad3 and decreasing its acetylation and transcription (160, 162, 163). In this way, RES was identified to ameliorate cardiac fibrosis by suppressing the TGF-β/Smad3 pathway by reducing Smad3 acetylation, which was mediated by promoting the activity of Sirt1 (101).

Autophagy and apoptosis are two crucial CH processes (164, 165). Various signaling pathways regulate these processes, and PI3K/AKT/mTOR pathway is one of them. Guan et al. investigated the preventive effects of RES treatment (30 mg/kg/day, 5 weeks) in male SD rats on autophagy and apoptosis in chronic intermittent hypoxia (CIH)-induced CH (110), which is mediated by decreasing Bax/Bcl-2 ratio, CIH-induced apoptosis, and oxidative stress, as well as increasing autophagy in rat models. Mechanistically, RES exerts its effects by suppressing PI3K/AKT/mTOR signaling pathway (110). Furthermore, PI3K/AKT/mTOR signaling pathway is suppressed by PTEN, while immunoproteasomes degrade PTEN. In this regard, RES (25 and 50 mg/kg/day, 2 weeks) was shown to partly protect cells from oxidative stress by inhibiting immunoproteasomes from degrading PTEN and eventually improving PO-induced CH in Male wild-type (WT) C57BL/6 mice (109). Immunoproteasomes are proteins involved in regulating immune responses, cellular metabolism, growth, and survival and consist of three immune subunits: β1i (LMP2/PSMB9), β2i (MECL-1/PSMΒ10), and β5i (LMP7/PSMB8). These subunits are associated with cardiac disorders (166, 167). A study by Chen et al. demonstrated that RES inhibited PTEN degradation by reducing the expression of immune subunits, which led to the activation of AMPK and inhibition of ATK/mTOR. In this study, RES treatment significantly alleviated cardiac hypertrophy and dysfunction. Treating Ang II-induced cardiomyocytes in-vitro with RES also decreased proteasome caspase-like, trypsin-like, and chymotrypsin-like activities, increased expression of PTEN, and decreased expression of cardiomyocyte size (109).

Oxidative stress inhibits LKB1 activity, leading to AMPK suppression (168). AMPK improves vascular function by activating endothelial NO synthase (eNOS) and producing nitric oxide (NO) (169, 170). In this light, RES was found to activate AMPK by enhancing the expression of LKB1, leading to increase NO production and improved vascular function. Indeed, Dolinsky et al. showed that RES (320 mg/kg/day, 5 weeks) prevents hypertension-induced hypertrophy by promoting the LKB1/AMPK/eNOS pathway and suppressing the p70S6k pathway in SHRs and C57BL/6 mice (90). Consistent with this study, Thandapilly et al. reported that RES (2.5 mg/kg/day, 10 weeks) improves CH and myocardial contractibility by exerting direct antioxidant effects on the heart in spontaneously hypertensive rats (SHRs) models (113). Inflammation, oxidative stress, and coronary artery endothelial dysfunction are essential in developing cardiac fibrosis (171). In a study by Zhang et al., treating HF mice with RES (10 mg/kg/day, 4 weeks) significantly reduced the cardiomyocytes' size and the heart weight-to-body weight (HW/BW) ratio, indicating that RES decreased CH. Functionally, RES suppressed cardiac inflammatory response by downregulating IL-1β, IL-6, and TNF-α and reducing inflammatory cell infiltration in cardiac tissue (101). Based on IL-10 production by macrophages involved in inducing myocardial fibrosis (172), the role of RES on cardiac macrophage activity was investigated. They found that the expression of M1 markers increased in the HF group. However, treatment with RES shifted macrophage polarization to the M2 phenotype, which has tissue repair effects (101). Atorvastatin is a lipid-lowering drug with anti-inflammatory, antioxidant, and antiproliferative properties (173, 174). Besides lowering cholesterol levels, atorvastatin has many beneficial effects on the cardiovascular system (175). Chakraborty et al. demonstrated that combination therapy with RES and atorvastatin (10 + 10 mg/kg/day, 25 days) in Wister Albino rats exerts more potent cardioprotective effects, compared with alone RES (20 mg/kg/day, 25 days) treatment, in terms of improved CH as well as decreased cardiac inflammation and oxidative stress (107).

#### 5.1.3. Resveratrol inhibits cardiomyocyte hypertrophy

CH is the pathological growth of the heart due to pressure or volume overload, loss of contractibility, chemical toxicity, and congenital disorders (176). CH is a beneficial adaptive response that removes the effect of extrinsic or intrinsic stress to preserve cardiac function. However, excessive CH is deleterious and leads to adverse complications such as HF (177). Cellularly, various transcription factors and signaling pathways, such as calcineurin-NFAT, PI3K/Akt/GSK-3, MAPK, and Gp130/STAT3 signaling pathways, participate in CH pathogenesis (178, 179). RES suppresses CH by regulating diverse signaling pathways in cardiomyocytes. Calcineurin is a calcium/calmodulin-activated serine-threonine phosphatase that plays a vital role in the development and progression of CH by regulating the nuclear factor of activated T cells (NFAT) phosphorylation. NFAT is a transcriptional factor that activates GATA4, which induces cell hypertrophy (180). The benefits of calcineurin inhibitors have been shown in terms of preventing CH (181). The study by Chan et al. showed that RES (0–100 µM, 24 h) inhibited NFAT transcriptional activity more efficiently than calcineurin inhibitors in neonatal rat cardiac myocyte cells, indicating an additional mechanism involved in the inhibitory effect of RES. Further examination revealed that RES inhibited CH processes by activating AMP-AMPK through upregulating liver kinase B1 (LKB1), the activating kinase of AMPK, leading to inhibition of p70S6K, eEF2, and NFAT signaling pathways (105). Also, AMPK could inhibit CH through NFAT, NF-κB, and MAPK signal pathways (182).

RES could exert beneficial effects by regulating micro RNAs (miRNAs), a central subgroup of non-coding RNAs, as an alternative molecular mechanism. Fan et al. showed that RES (150 mg/kg, 4 weeks) improves cardiac function and ameliorates CH by inhibiting the expression of miR-155 through upregulating BRCA1 in mice models of cardiac hypertrophy. They showed that miR-155 induces cardiomyocyte hypertrophy and disrupts cardiac function (111). BRCA-1 is an essential gene for heart development and exerts cardioprotective effects in adult hearts (183). Angiotensin II is one of the renin-angiotensin system hormones with a vital role in the progression and development of several CVDs, such as hypertension, CH, and HF (184). Mashhadi et al. showed that expression of AT1a (Ang receptor) was increased in the hypertrophic rat heart, indicating that the Ang receptor plays a vital role in the development of PO-induced cell hypertrophy. It has been demonstrated that RES (1 mg/kg/day, 35 days) decreased the expression of AT1a mRNA in the hypertension group of Male Wistar rats, indicating that RES prevents PO-induced CH by decreasing the expression of AT1a (112). Moreover, it was observed that treatment of RES (10–50 mg/kg/day, 4 weeks) in the partially nephrectomized rat models was effective in nephrectomy-induced hypertension and the subsequent CH by reducing NO, AngII, and ET-1 concentrations, which are hypertrophic cell agents (91).

#### 5.1.4. Resveratrol can improve exercise capacity in Hf mice

Exercise intolerance is an essential feature of chronic HF, and identifying its underlying mechanism may help improve the quality of life of patients with HF (185). Recent studies have shown that impaired skeletal muscle function, structure, and metabolism play an important role in exercise intolerance in HF patients (186). In this way, some studies have shown that RES can improve rodents' skeletal muscle biogenesis, isometric force, and metabolism (187, 188). Sung et al. in 2015 showed that RES (320 mg/kg/day, 2 weeks) improved the exercise capacity of HF C57Bl/6N mice by increasing the flow-mediated vasodilatation and vascular function (19). A further study investigated the treatment effect of 450 mg/kg/day RES for two weeks on improving exercise intolerance of C57Bl/6N mice with pressure overload-induced HF. A significant improvement in HF mice's respiratory exchange ratio (RER) was observed. Decreased RER in HF mice indicates more use of fatty acids than glucose in metabolism, producing more ROS and increasing oxidative stress. Treatment with RES elevated RER level, indicating that substrate utilization in the metabolic cycle shifted towards glucose; also, the total metabolic rate was increased after RES treatment (102). Mechanistically, RES treatment increased phosphorylation and expression of IRS-1, Akt, and AMPKα, which were reduced in HF and enhanced insulin sensitivity of skeletal muscles. This study also indicated that RES improved metabolism and insulin resistance in HF mice by altering the gut microbial community, which is associated with systemic metabolic rate and insulin sensitivity. Sung et al. showed that RES enhanced the cecum bacterial profile, increasing glucose homeostasis and carbohydrate metabolism (102).

### 5.2. Curcumin

Curcumin [(1E,6E)-1,7-bis(4-hydroxy-3-methoxyphenyl) hepta-1,6-diene-3,5-dione] is a natural polyphenol substance extracted from turmeric that plays substantial roles in cellular function by regulating various signaling pathway. Curcumin benefits various pathologic conditions and has anti-inflammatory and antioxidant effects (189). Various clinical and pre-clinical investigations have revealed Curcumin is a safe natural polyphenol compound without any severe adverse effects in a dose of 12 g/day for human subjects (190). Moreover, EFSA stated acceptable daily intake of curcumin is about 3 mg/kg in healthy individuals (191). According to these features, curcumin can be used as a therapeutic element in various diseases (192). Several studies have reported the positive role of curcumin in attenuating CVDs, such as atherosclerosis, MI, and HF (193, 194). Tang et al. studied the effects of curcumin (100 mg/kg/day, 8 weeks) on the cardiac function and structure of rabbits with chronic heart failure. The results demonstrated the ameliorating of curcumin's role in cardiac remodeling and HF's diastolic dysfunction. Also, curcumin significantly reduced collagen deposition in heart tissue (114) (Table 1).

#### 5.2.1 Curcumin inhibits p300 histone acetyltransferase activity

The p300, a histone acetyltransferase (HAT) enzyme, regulates the expression of multiple genes by chromatin remodeling. P300 can also coactivate other transcriptional factors, such as GATA4 (195, 196), a member of the zinc-finger transcription factor family with high expression in cardiomyocytes and plays a critical role in cardiomyocyte differentiation (197). GATA4 regulates the transcription of ANP, α- and β-myosin heavy chain (α-MHC and β-MHC), which play a role in cardiac remodeling (198). Therefore, it seems that overexpression of p300 induces cardiac remodeling by increasing the activation of GATA4 (199). Several studies have reported curcumin as a natural p300-specific HAT inhibitor that can improve cardiac remodeling in HF. Morimoto et al. *in vitro* and *in vivo* studies exhibited the promising effect of curcumin (50 mg/kg/day, 7 weeks) as a p300 inhibitor on preventing cardiac remodeling in both MI-induced HF and hypertensive rats with HF (116). Mechanistically, curcumin reduced GATA4 and p300 binding but did not change their expression. Indeed, curcumin reverses cardiac remodeling and hypertrophy by inhibiting p300 histone acetyltransferase activity and decreasing the acetylated form of GATA4 and p300/GATA4 complex (116).

#### 5.2.2. Curcumin exerts cardioprotective effects by regulating DDK-3

Dickkopf-related protein 3 (DKK-3), a member of dickkopf glycoprotein, regulates cell proliferation. Also, DKK-3 could modulate the immune system by acting as a cytokine-like protein (200, 201). DDK-3 is involved in cardiac remodeling and vascular smooth muscle differentiation, and several studies demonstrated the cardioprotective effects of DDK-3 (202). Zhang et al. showed that suppressing the expression of DDK-3 increases cardiac dysfunction and remodeling while augmenting the expression of DDK-3 improves cardiac function in an animal model of HFrEF (203). Moreover, DDK-3 improves chronic HF conditions by suppressing c-Jun N-terminal kinase (JNK) signaling pathways by inhibiting p38 mitogen-activated protein kinase (p38) (204). It has been found that P38 and JNK signaling pathways are highly activated in HF and participate in cardiac remodeling (205, 206). In an intriguing study, treating HF mice with curcumin for ten weeks significantly improved cardiac function, remodeling, and decreased expression of biomarkers related to them_ ROS, TNF-α, MMP-2, and MMP-9. In addition, expression of DDK-3 increased in curcumin-treated mice, compared with untreated mice with HF. Curcumin treatment (100 mg/day/kg, 10 weeks) in rabbits with chronic heart failure was associated with decreased p38/JNK signaling pathway activation and expression of ASK1, one of the upstream components of these pathways. So, these results indicate that curcumin exerts cardioprotective effects by increasing DDK-3 and ASK1 expression, suppressing the p38/JNK signaling pathway (115).

#### 5.2.3. Curcumin and other mechanisms

Liu et al. studied the effects of curcumin on isoproterenol (ISO)-induced CH and the molecular mechanism behind it. The result showed that curcumin (200 mg/kg/day, 4 weeks) prevented ISO-induced CH in Male Sprague-Dawley rats and cardiac fibrosis by decreasing autophagy through positively modulating mTOR (117). It has been found that the mTOR signaling pathway exerted cardioprotective effects by decreasing autophagy (207). Consistent with these studies, Bai et al. also showed that curcumin treatment (50 mg/kg/day, 9 weeks) prevented CH and improved cardiac function in male Wistar rats. Also, curcumin improved vascular relaxation by increasing endothelium response to acetylcholine and increasing expression of the Na^+^/Ca^2+^ exchanger (NCX) and eNOS in the myocardium and vascular endothelium. Treatment with NCX inhibitor KB-R7943 reversed the protective effects of curcumin on the myocardium and vessels. These results indicate that curcumin exerts cardioprotective effects by upregulating NCX expression in response to increased afterload (119).

### 5.3. Quercetin

Quercetin (QCT) is a natural flavonoid polyphenol widely found in fruits and vegetables such as onions, peppers, plums, mangos, and berries and is a potent anti-inflammatory, antioxidant, and anti-cancer component (208–211). Quercetin regulates several molecular pathways in cellular processes and exerts many beneficial effects on the cardiovascular system, such as anti-hypertensive and cardioprotective effects, by suppressing inflammation, oxidative stress, and apoptosis in the heart (212, 213). Several studies investigated the mechanisms behind the cardioprotective effects of QCT. Liu et al. showed that QCT inhibits the NF-κB pathway by activating peroxisome proliferator-activated receptor-γ (PPARγ) protein and preventing cardiac damage (214). Moreover, a recent study showed that QCT protects cardiomyocytes against oxidative stress by regulating mitophagy and endoplasmic reticulum stress by suppressing the SIRT1/TMBIM6 pathway (215).

For further investigation, Chang et al. administered QCT to improve cardiac function and hypertrophy in rats with TAC-induced HF. QCT decreased cardiac fibrosis by modulating TGF-β and MMPs, inflammatory cytokines (TNF-α, IL-13, IL-18), and production of ROS by activating the expression of SIRT5 and IDH2 desuccinylation. Succinylation regulates mitochondrial metabolism and function; impaired succinylation and mitochondrial function lead to oxidative stress and inflammation. This study showed that QCT regulates mitochondrial energy metabolism and improves oxidative stress by increasing succinylation through increasing expression of SIRT5 (216). Anti-hypertrophic properties of quercetin have been reported in several studies (217–219). QCT can prevent cardiac remodeling in mice. Wang et al. showed that QCT decreased cardiomyocyte Ca^2+^ oscillation frequency, resulting in regulated excitation-contraction of the myocardium and anti-arrhythmia effects. Also, quercetin attenuated ventricular wall thickness in mice, indicating the anti-hypertrophic effects of QCT (219). Additionally, QCT treatment (20 mg/kg/day, 8–12 weeks) in Spontaneous hypertensive rats improved CH and cardiac fibrosis by preventing mitochondrial dysfunction. SIRT3/PARP-1 was this study's suggested target pathway of QCT (120). SIRT3 is a type III histone deacetylase with anti-hypertrophic effects on the heart (220). The expression of SIRT3 was increased by QCT, indicating that the anti-hypertrophic effects of QCT are related to the SIRT3-mediated signaling pathways. PARP-1 is an up-regulated enzyme in hypertrophic hearts, and overexpression of SIRT3 inhibits its function (220). Moreover, In vitro investigation showed that treating Ang II-induced H9c2 cells with QCT prevents hypertrophic response, restores impaired mitochondrial structure and function, reduces ROS generation, and decreases oxidative stress (120).

In another study, QCT treatment (10 mg/kg/day, 4 days) reversed ISO-induced CH in male swiss mice by conducting antioxidant effects and improving mitochondrial dysfunction (121). Oxidative stress and ROS play essential roles in the development of CH. In this study, QCT treatment restored endogenous antioxidant enzyme (CAT and SOD) activity, increased sulfhydryl protein levels, and decreased H_2_O_2_ (a major ROS). Ca^2+^-induced swelling of mitochondria in ISO rats reduced after treatment with QCT; activating the mitochondrial SOD enzyme and increasing resistance toward Ca^2+^-induced swelling are the possible mechanisms. Altogether this study revealed that QCT attenuates pre-existing CH by balancing oxidation and protecting mitochondria (121). QCT (130 mg/kg/day, 7 days) also reduced mean arterial blood pressure and aortic medial wall thickening, improved cardiac function, and prevented cardiac hypertrophy in PO-induced male Sprague-Dawley rats by abdominal aortic constriction (AAC) (221). Protein kinase C (PKC), extracellular regulated kinase 1/2 (ERK1/2), and Akt are contributing signaling pathways in CH pathogenesis. In this study, treatment with QCT normalized PKCβII translocation and inhibited PKC and ERK1/2 pathways in AAC rats. QCT also reduced oxidative stress in the heart tissue of AAC rats. This study showed that rats with a QCT-supplemented diet have lower blood pressure and CH in pressure-overload stress conditions (221) (Table 1).

### 5.4. Gallic acid

Gallic acid (3, 4, 5-trihydroxy benzoic acid) is a polyphenol component in green tea, blackberry, grapes, wine, mangoes, and walnuts. It can be an anti-cancer, anti-allergic, anti-microbial, antioxidant, and anti-inflammatory factor (222, 223). Gallic acid (GA) impacts the cardiovascular system, such as attenuating cardiac fibrosis, hypertension, oxidative stress, and eventually HF. The antioxidative effects of gallic acid suppress cardiac hypertrophy and protect cardiomyocytes against damage (127, 224, 225). An *in vitro* investigation conducted by Yan et al. showed that GA suppresses angiotensin II (Ang II)-induced cardiomyocytes hypertrophy by inhibiting Ang II-related pathways such as JAK2, p-STAT3, p-ERK1/2, p-AKT, and NFATc1 and reducing expression of Ang II downstream products (GFR, gp130, and CaNA). In this experiment, GA increased cardiomyocyte autophagy by inhibiting ULK1 phosphorylation (autophagy regulator), which leads to the degradation of unnecessary products and improved cardiac remodeling. GA at 1–200 μM had no significant toxic effect on cardiomyocytes. On the other hand, *in vivo* study revealed that treated TAC-induced HF C57BL/6 mice with GA (5 or 20 mg/kg, 8 weeks) led to downregulating the expression of ANP, BNP, and β-MHC, thereby decreasing CH. GA decreased inflammatory markers, myocardial superoxide products, and fibrosis factors in the heart of mice with HF. Collectively, GA exerts its cardioprotective effects and improves cardiac remodeling by activating autophagy through various pathways (126). In another study, GA (100 mg/kg/day, 2 weeks) significantly improved cardiac size and cardiac function in TAC-induced HF CD-1 male mice models, compared to conventional drugs of HF. The levels of HF markers, such as ANP, BNP, and β-MHC, significantly decreased after GA treatment. Further investigation revealed that GA reduced cardiac fibrosis by decreasing the phosphorylation of Smad3 protein and inhibiting the TGF-β1/Smad signaling pathway (127).

Pulmonary fibrosis is a severe complication of HF due to increased left atrial pressure, pulmonary edema, and fibrosis formation (226). According to the anti-fibrotic effects of gallic acid in other studies, Jin et al. found that treatment of TAC-induced HF CD-1 male mice with GA (100 mg/kg/day, 2 weeks) reduced pulmonary fibrosis by downregulating the expression of collagen type I, fibronectin, and connective tissue growth factor (CTGF). Expression of epithelial-mesenchymal transition (EMT) markers, including N-cadherin and SNAI1, was increased in the TAC group, and treatment with GA reduced their expression, suggesting the inhibitory role of GA in HF-induced pulmonary fibrosis by inhibiting the EMT process (128). EMT participates in tissue repair and induces pulmonary fibrosis (227). According to the well-established effects of GA on CVDs, Jin et al. investigated mechanisms of GA affecting blood pressure and CH. Treatment with GA (320 mg/kg/day, 16 weeks) lowered systolic blood pressure and suppressed the RAAS system by decreasing aortic ACE1 and AT1 receptor levels in Wistar-Kyoto rats owning hypertension-induced cardiac hypertrophy. Arterial remodeling and vascular contractibility were also reduced by GA treatment, indicating the regulatory effect of GA on vascular smooth muscle. Collectively, GA alleviated hypertension and CH by suppressing Nox2 activity and Nox2-induced oxidative stress via inhibiting GATA4 expression (78).

Additionally, GA prevented ISO-induced CH and improved cardiac dysfunction by decreasing the expression of hypertrophy-related and fibrosis-related genes. GA (100 mg/kg/day, 3 weeks) suppressed the JNK2/ERK1/2 signaling axis (members of the MAPK pathway) in Male CD-1(ICR) mice with ISO-induced CH. To investigate the role of JNK2 in cardiac fibrosis and hypertrophy, they transfected JNK2 into cardiomyocytes which led to increased Smad3 and collagen type I protein levels, inducing cell hypertrophy and fibrosis. Consequently, this study showed that GA prevents ISO-induced cardiac hypertrophy by regulating JNK2 signaling and Smad3 binding activity (129) (Table 1).

### 5.5. Genistein

Estrogen has shown an influential role in protecting the cardiovascular system, especially in improving the pulmonary and cardiac function of pulmonary hypertension (PH) cases, by binding to estrogen receptor-β (ERβ) (228, 229). Genistein, an isoflavone derived from soybean, functions as a natural estrogen, can bind to ERβ stronger than estrogen, and can exert anti-inflammatory and anti-cancer effects (230–232). According to the unwanted side effects of estrogen as a drug, genistein can be used instead of the pharmacological form of estrogen. Also, several studies have shown that genistein intake can ameliorate cardiovascular risk factors such as hypertension and lipid profile (233). Matori et al. showed that treating the PH Male Sprague-Dawley rats models with 1 mg/kg/day genistein attenuated RV remodeling, PH-induced RHF, lung remodeling, and pulmonary fibrosis. An *in vitro* examination demonstrated that genistein (1 μmol/l, 48 h) suppressed the proliferation of human pulmonary artery smooth muscle cells and inhibited cardiomyocyte hypertrophy by binding to the ER-β receptor. This study confirms the beneficial cardiopulmonary effects of genistein in rats with PH (130).

Genistein has shown anti-hypertrophic effects in several studies. Meng et al. reported that genistein (40 mg/kg/day, 7 weeks) attenuates PO-induced CH in C57/BL6 male mice and improves cardiac function by directly decreasing phosphorylation JNK1/2, thereby blocking this signaling pathway (131). Nitric oxide synthases (NOS) catalyze NO production from L-arginine. Neuronal nitric oxide synthase (nNOS) and endothelial nitric oxide synthase (eNOS) have anti-hypertrophic effects on the heart, but inducible nitric oxide synthase (iNOS) induces hypertrophy (234). Maulik et al. investigated the effects of genistein (0.1 and 0.2 mg/kg/day, 14 days) on ISO-induced CH in male Wistar rats and investigated the role of NOS enzymes in developing cardiac hypertrophy. Genistein exerts anti-hypertrophic effects via eNOS, nNOS, and antioxidant effects in ISO-induced CH rat models (132). Recent studies have shown that genistein exerts parts of its effects by regulating the expression of miRNAs (235). Gan et al. showed that treatment of female ICR mice with genistein (100 mg/kg/day, 21 days) upregulated miR-451, suggesting that genistein exerts its anti-hypertrophic effects via enhancing expression of miR-451 and miR-451 suppresses cardiac hypertrophy by directly inhibiting TIMP2 expression. Expression of the TIMP2 gene was increased in the ISO-induced CH heart, indicating pro-hypertrophic effects of TIMP2 (133) (Table 1).

### 5.6. Pterostilbene

Pterostilbene [4-(3,5dimethoxystyryl) phenol] is a polyphenol structurally similar to RES and has potential pharmacological impacts such as anti-cancer, anti-inflammatory, antioxidant, and anti-apoptosis (236–238). Similar to RES, Pterostilbene (PTS) also exerts cardioprotective effects by suppressing oxidative stress and apoptosis by activating the AMPK pathway (239) or by upregulating PGC1α through activating AMPK/SIRT1 pathway (240). Furthermore, Lacerda et al. revealed that administration of PTS-HPβCD complex (25, 50, or 100 mg/kg/day, 2 weeks) prevents right ventricular (RV) remodeling and improves RV function in pulmonary hypertension-induced right-sided HF rat models. Enhancing the glutathione redox cycle was suggested as an underlying mechanism of the antioxidative effects of PTS (134). The glutathione redox cycle protects the cell membrane from LPO and oxidative stress (241). Also, expression of total phospholamban (PLB) and SERCA (sarco/endoplasmic reticulum Ca^2+^-ATPase) was increased after treatment with PTS-HPβCD complex, indicating that PTS improves cardiac contractibility and function by improving the calcium handling process in the heart (134). Phospholamban is a protein that regulates calcium channels in the heart, and SERCA is a calcium transporter in the cell. Both these proteins play a significant role in calcium transportation and cardiac muscle contractility (242, 243) (Table 1).

### 5.7. Kaempferol (KF)

Kaempferol (3,4′,5,7-tetrahydroxyflavone) (KF) is a type of flavonoid found in grapes, tomatoes, aloe Vera, *coccinia grandis*, and *moringa oleifera* (244). Kaempferol can protect cells against oxidative stress and inflammation and exerts various pharmacological effects such as anti-microbial, anti-diabetic, and anti-cancer (245). Kaempferol exerts pleiotropic beneficial effects on the cardiovascular system, including ameliorating cardiac fibrosis, preventing HF, improving myocardial damage repair, and decreasing atherosclerosis (135, 246, 247). Zhou et al. showed that KF (15 mmol/l, 10 min) improves myocardial ischemia by exerting antioxidant activity and inhibiting glycogen synthase kinase-3 beta (GSK-3β) activity in adult male Sprague-Dawley rats with myocardial ischemia/reperfusion (I/R) injury (247). Diabetes is one of the significant risk factors for HF development (248); thus, according to the positive effects of KF on both the cardiovascular system and diabetes, Zhang et al. investigated the effect of KF (20 mg/kg/day, 42 days) in diabetic adult male Wistar rats with HF. Results of this study demonstrated that treatment with KF exerts significant cardioprotective effects in diabetic rats as it suppresses inflammation, oxidative stress, and apoptosis and alleviates blood glucose levels and serum cardiac markers (135).

Regarding the underlying mechanism, KF modulated the nuclear factor erythroid 2-related factor 2 (Nrf2) signaling pathway, which protects cells against ROS, and the nuclear factor-light-chain-enhancer of activated B cells (NF-κβ) signaling pathway, which has a vital role in fibrosis. The activity of antioxidant enzymes was increased, while the LPO level was decreased after treatment with KF. Expression of inflammatory factors, including NF-κβ p65, TNF-α, IL-6, IL-1β, p-IKKβ, and COX-2, significantly decreased. KF enhanced AKT/GSK-3β singling pathway by increasing the phosphorylation of AKT and GSK-3β. Decreased phosphorylation of p-p38 MAPK and decreased expression of PI3K in the KF group indicates the inhibitory role of KF on ERK/p38 MAPK and PI3K signaling pathways. Cardiac apoptosis markers (caspase-3 and Bax) were decreased, while the anti-apoptotic marker (Bcl-2) was increased in cardiac tissue after KF treatment. The histopathological investigation revealed decreased destruction of myofibrils and lesser TUNEL-positive cells in the cardiac tissue of rats treated with KF (135).

Feng et al. investigated the role of KF in CH and the molecular mechanism behind it. Treatment with KF (100 mg/kg/day, 6 weeks) improved cardiac function and prevented CH in mice that underwent aorta banding surgery. Interstitial fibrosis, oxidative stress, and apoptosis were also reduced after KF administration (141). ASK1/MAPK and their downstream targets, p38, and JNK were upregulated in the PO-induced CH condition. The stimulating activity of ASK1/MAPK signaling pathways in H9c2 cardiomyocytes *in vitro* enhanced cardiomyocyte growth and enlargement, whereas treatment with KF reversed these results. Therefore, it could be speculated that KF functioned by reducing oxidative stress and suppressing apoptosis by inhibiting ASK1/MAPK signaling pathway (141). ASK1/MAPK signaling pathway plays a pivotal role in response to stress, especially oxidative stress, and induces apoptosis (249). P38 and JNK are subfamilies of MAPKs and regulate cell apoptosis (250) (Table 1).

### 5.8. Luteolin

Luteolin (3′,4′,5′,7′-tetrahydroxyflavone) (LUT) is a type of flavonoid found in carrot, cabbage, tea, celery, and apple that possess various beneficial effects such as antioxidant, anti-inflammatory, and anti-apoptotic (251). Recent studies have reported that LUT exerts cardioprotective effects against myocardial ischemia, coronary artery disease, and HF (136, 251). Hu et al. reported that treating HF Sprague-Dawley rats with LUT (10 ug/kg/day, 10 days) improves cardiomyocyte contractibility and heart function and decreases apoptosis and cardiac fibrosis. LUT increases cardiac contractibility by increasing intracellular Ca^2+^ (136). Sarcoplasmic reticulum Ca^2+^-ATPase 2a (SERCA2a) regulates excitation/contraction coupling in the heart by regulating the concentration of Ca^2+^ and has been shown to play an essential role in HF pathogenesis as its expression decrease in HF (252, 253). PLB is a SERCA2a inhibitor whose phosphorylation suppresses activity (254). Hu et al. showed that LUT increased the expression of SERCA2a and Sp1, a transcription factor, by activating the PI3K/Akt pathway and increasing PLB phosphorylation. This study verified the enhancing role of LUT on cardiac contractibility in HF (136) (Table 1).

### 5.9. Epigallocatechin gallate (EGCG)

Epigallocatechin gallate (EGCG) is a natural polyphenol flavonoid abundant in green tea and is known for its antioxidant properties. Several studies have shown that EGCG is vital in decreasing oxidative stress in the cardiovascular system (255). For instance, a study by Sheng et al. showed that EGCG attenuates PO-induced CH by suppressing apoptosis and oxidative stress in rats (256). Another study investigated the possible mechanism of EGCG (50, 100 mg/kg/day, 6 weeks) in attenuating PO-induced CH in Male Sprague–Dawley rats. The findings implied the role of EGCG in reducing oxidative stress and apoptosis; EGCG increased the expression of Bcl-2 and telomere repeat-binding factor 2 (TRF2), which are anti-apoptotic agents. In addition, EGCG reduces oxidative stress in the heart by decreasing the level of MDA and increasing SOD activity (140). According to the cardioprotective effects of EGCG, Muhammad et al. showed the beneficial effects of EGCG (200 mg/kg/day, 30 days) in preventing aging-mediated CH, fibrosis, and remodeling, as well as in improving cardiac function in aged Wistar albino rats. Mechanistically, EGCG decreased the production of ROS, improved the antioxidant defense system, and reduced apoptosis via suppressing TGFβ/TNFα/NF-κB pathway in (139) (Table 1).

### 5.10. Apigenin

Apigenin (4′,5,7-trihydroxyflavone) is a flavonoid component distributed in various plants like parsley, oranges, and garlic and has different beneficial pharmacological effects, including antioxidant and anti-inflammatory effects (257). In this regard, Gao et al. investigated the effect of apigenin treatment on improving hypertension and hypertension-induced CH in Wistar-Kyoto rats. They showed that apigenin (20 μg/h, 4 weeks) prevents cardiac hypertrophy via suppressing NADPH oxidase-dependent ROS (137). Previous studies have reported that hypoxia-inducible factor (HIF)-1α mediates CH through its effect on HIMF (hypoxia-induced mitogenic factor) (38, 258). Apigenin can exert inhibitory effects on HIF-1α; therefore, Zhu et al. investigated the anti-hypertrophic effects of apigenin by inhibiting HIF-1α. They treated cardiac hypertrophy induced by renovascular hypertension in rats with apigenin (50–100 mg/kg/day, 4 weeks). The result showed that apigenin decreased blood pressure, heart weight, and serum angiotensin II, indicating a positive role of apigenin in hypertension and CH. Levels of serum and myocardial-free fatty acids also decreased after apigenin treatment. HIF-1α deregulates cardiac metabolism by inhibiting the expression of mitochondrial proteins involved in metabolisms, such as PPARα, CPT-1, and PDK-4. Apigenin improved abnormal myocardial glucolipid metabolism by upregulating PPARα, CPT-1, and PDK-4 via suppressing HIF-1α. This study revealed that apigenin's anti-hypertrophic effects are associated with its effect on HIF-1α (138) (Table 1).

### 5.11. Caffeic acid

Caffeic acid (CA) is a natural flavonoid found in various foods and herbs, such as coffee, red wine, thyme, sage, and spearmint (259). CA has a wide range of beneficial effects on cellular processes, such as modulating cell growth, proliferation, and anti-inflammatory effects; hence, this molecule could positively affect the cardiovascular system. It is reported that intravenous administration of caffeic acid decreases blood pressure and improves cardiac function (260). It also exerts cardioprotective effects by reducing MI-related oxidative stress (261). According to the association of CA with the MAPK pathway and the role of this pathway in CH, Ren et al. indicated inhibitory effects of CA on PO-induced CH. In addition, the treatment improved cardiac function and decreased cardiac fibrosis. CA treatment (100 mg/kg/day, 6 weeks) in the C57 male mice with induced cardiac hypertrophy downregulated the MAPK pathway by decreasing phosphorylation of ERK1/2, indicating that MAPK suppression mediates CA anti-hypertrophic effects. Consistent with these results, CA suppressed PE-induced hypertrophy *in vitro* by downregulating MEK/ERK signaling pathway (143) (Table 1).

### 5.12. Delphinidin

Delphinidin (3,3′,4′,5,5′,7-hexahydroxyflavylium) is a flavonoid anthocyanidin found in pigmented fruits and vegetables, such as blueberry. The anti-inflammatory and antioxidant effects of delphinidin were well described (262). ROS is a significant mediator of oxidative stress and has a vital role in the development of CH by regulating various signaling pathways and protein kinases (263). NADPH oxidase (NOX) is an oxidoreductase enzyme and the leading producer of ROS in cardiac myocytes (264, 265). Previous studies have shown that delphinidin exerts parts of its function by regulating NOX activity. Chen et al. showed that treatment with a high dosage of delphinidin ameliorated CH and cardiac dysfunction and reduced oxidative stress and cardiac fibrosis in the heart. In vitro, delphinidin (50 μM, 24 h) decreased the increased myocardial NOX activity after Ang II induction; reduced ROS production; and prevented cardiomyocyte hypertrophy in Neonatal rat cardiomyocytes, with no cytotoxic effects (142). Treatment with delphinidin prevented the increased expression of NOX by activating AMPK, a NOX inhibitor, in response to hypertrophic stimulators. Furthermore, high-dosage delphinidin treatment increased the expression of Erk1/2, Jnk1/2, and p38 (kinases of MAPK signaling pathway), indicating the role of the MAPK signaling pathway in the anti-hypertrophy effect of delphinidin. Exploring the effects of delphinidin (15 mg/kg/day, 8 weeks) on aged-mediated cardiac remodeling showed that delphinidin reversed CH by suppressing the phosphorylation of AMPK and the activity of NOX in C57BL/6 mice with the aging-related cardiac hypertrophy (142) (Table 1).

### 5.13. Hesperidin

Hesperidin (3,5,7-trihydroxyflavanone 7-rhamnoglucoside) (HES) is a natural flavonoid with comprehensive pharmacological properties such as anti-inflammatory and antioxidant (266). HES has beneficial cardioprotective effects, especially in attenuating CH (267). A recent study revealed that HES (200 mg/kg/day, 28 days) alleviates ISO-induced CH by suppressing oxidative stress, apoptosis, and inflammation in CH-induced male albino Wistar rats. It was indicated that treatment with HES improved hemodynamic state and attenuated left ventricular function. HES preserved the function and structure of mitochondria, myofibril, and myocyte. Cardiac injury, apoptosis, and inflammatory markers were decreased, and oxidative stress was reduced in heart tissue by HES treatment. PPAR-γ is a metabolism regulator which suppresses CH. Bhargava et al. showed that HES upregulated the expression of PPAR-γ, indicating that HES anti-hypertrophic effects arise from enhancing PPAR-γ expression (144) (Table 1).

## 6. Polyphenols and bioavailability

Bioavailability is an essential issue regarding the clinical application of pharmacological substances. Although RES is reported as a safe component with no adverse effects on humans, its low bioavailability (about 20%) is an essential drawback of using it as a drug. The concentration of RES is dose-depended, and the maximal concentration of 2.4 µM results from the administration of 5 g RES. Drug delivery systems and reformed formulation are novel ways to improve the bioavailability of RES in humans. RES has been shown to improve myocardial reperfusion, enhancing re-endothelialization and reducing inflammation. Also, RES consumption before MI reduced infarct size and cardiac arrhythmia and relieved myocardial injury faster. RES can increase the viability and proliferation of cardiac stem cells, therefor transplanting RES-treated stem cells into an ischemic heart improved cardiac injury in the peri-infarct zone (268).

While curcumin could be administered as a drug, pure curcumin has low oral bioavailability due to its low absorption and rapid metabolism. In this regard, Ray et al. increased curcumin bioavailability by designing a novel delivery system; they encapsulated curcumin within the carboxymethyl chitosan (CMC) nanoparticle and conjugated it to a myocyte-specific homing peptide (CMC/peptide). This agent's 5 mg/kg showed better bioavailability than 35 mg/kg of pure curcumin. Treatment with low dose CMC/peptide downregulated expression of hypertrophic markers, apoptotic markers, and mediators. Collectively, Ray et al. showed that an efficiently targeted delivery regimen for curcumin enhances its therapeutic effects, reduces CH, and improves cardiac function (269).

Additionally, Sungawa et al. investigated the effectiveness of a novel surfactant-soluble oral drug delivery system (DDS) for curcumin to develop a therapeutic aid for patients with MI-induced HF. This DDS significantly enhanced gastrointestinal absorption and effectiveness of curcumin, as administration of 0.5 mg/kg of DDS curcumin exerted the same effect as 50 mg/kg of pure curcumin. In the meantime, no significant side effects were detected regarding DDS curcumin administration. The histopathologic examination showed that DDS curcumin suppressed myocardial cell hypertrophy and perivascular fibrosis. However, the exact dosage of pure oral curcumin did not apply the same effects (270).

Creating a synthetic analog for curcumin is another way to increase its bioavailability to use it as an efficient therapeutic drug. Shimizu et al. examined the role of five different curcumin analogs in inhibiting p300-HAT. Among these five analogues, GO-Y030 ((1E, 4E)-1,5-bis[3,5-bis(methoxymethoxy)phenyl]-1,4-pentadiene-3-one) inhibited p300-HAT and improved HF more efficiently than curcumin. Treating epinephrin-induced cardiomyocyte with GO-Y030 at 1/10th of the curcumin dose (1 μM vs. 10 µM) suppresses the interaction between p300 and GATA4. The in-vivo investigation conducted by Shimizu et al. revealed that administration of 0.5 mg/kg of GO-Y030 prevented TAC-induced HF, CH, and cardiac fibrosis significantly, to the same extent as 50 mg/kg/day curcumin. GO-Y030, at a dose 1/100th that of curcumin, collectively improves HF and can be effective clinically in treating HF without notable liver and renal toxicity (271).

## 7. Polyphenols and clinical trials

HF treatment is based on improving symptoms and preventing further complications. Although these treatments effectively relieve symptoms, the survival rate has not met the desirable goal yet. Therefore, recent studies and clinical trials have focused on targeting the pathogenic mechanism of HF (such as myocardial contractibility and metabolism, inflammation, oxidative stress, etc.) with new drugs (272, 273). Therefore, Gal et al. investigated the role of RES on left ventricular function and cardiac inflammation in patients with HF. In this randomized, double-blind study, 3-month treatment with 100 mg/day RES significantly reduced levels of total cholesterol, LDL-cholesterol, and inflammatory cytokines (IL -1 and IL -6).

Moreover, the levels of NT-proBNP (a marker of the severity of HF) and galectin-3 were significantly lower in the RES-treated group compared to the placebo group. Echocardiography results showed that treatment with RES significantly increased ejection fraction, left ventricular stroke volume, left ventricular end-systolic volume, and ventricular dilation, indicating positive effects of RES on cardiac remodeling. The results showed that RES suppressed the expression of ATP synthesis-related genes via oxidative phosphorylation in leukocytes; however, this did not result in mitochondrial dysfunction. This clinical study demonstrated that RES has anti-inflammatory effects and improves the quality of life, physical performance, cardiac function, and remodeling in patients with MI-induced HF (99).

In another similar study, Gal et al. evaluated the effects of RES on hemorheological parameters in HF patients. They found that RES could significantly improve impaired red blood cell (RBC) aggregation resulting from HF, which may be attributed to the antioxidative effects of RES. Also, the result showed that RES significantly improved the results of the 6-minute walk test of HF patients (274). Various cardiovascular disorders are associated with increased RBC aggregation, such as ischemic heart disease, diabetes, venous thrombosis, and HF (275). Increased levels of inflammatory cytokines and oxidants in the blood due to HF are significant factors in RBC aggregation and hemorheological disturbances (276, 277). Unstable angina, a subtype of acute coronary syndrome (ACS), leads to cardiac complications, such as arrhythmia, MI, and eventually HF. Inflammation is a critical factor in the progression of ACS, leading to further complications. According to the anti-inflammatory effects of curcumin, it can be used as a therapeutic drug in ACS, preventing complications such as HF. Dastani et al. investigated the effects of administrating 80 mg/day of curcumin to patients with unstable angina in a randomized, double-blind clinical trial. This novel clinical trial showed no significant effect of curcumin on preventing cardiovascular complications of unstable angina, such as HF (278). In a double-blind, placebo-controlled, randomized clinical trial, the effect of high-dose of curcumin (a 90 mg curcumin capsule, twice a day for 24 weeks) on the prevention of hypertensive heart disease was investigated. The results indicated that high curcumin absorption had no significant effects on left ventricular diastolic function but significantly suppressed increment in the plasma BNP levels (279). In another double-blind, placebo-controlled, randomized clinical study, the efficiency of nano-curcumin (80 mg/day) consumption on cardiovascular risk factors in patients with type diabetics as well as mild to moderate coronary artery disease. The result revealed that nano-curcumin reduced atherosclerosis and hs-CRP levels as an inflammation indicator in diabetic heart patients (280). However, future studies still need to investigate the theory more widely.

## 8. Future perspective

As reviewed earlier, polyphenols can play a critical role in attenuating HF and CH, as they can prevent cardiac remodeling mechanistically by blocking oxidative, inflammatory, apoptotic, and fibrotic-related pathways. Moreover, polyphenols are easily accessible and enriched within various natural plant-based sources and could be considered novel promising therapeutic approaches for HF. Additionally, the conventional HF treatments' low survival rates have forced studies to search for other viable options as CVDs are growing globally. However, these studies are still in their infancy, and limited clinical data regarding the efficacy, side effects, and administration routes of polyphenol-based drugs for treating cardiovascular diseases such as HF and CH are available.

Despite the abundant presence of polyphenols in a human's regular nutritional diet, the poor solubility, low stability, rapid metabolism and elimination, and eventually, weak bioavailability are the rate-limiting factors for inhibiting these compounds from exerting their full cardioprotective effects (281). Therefore, some recent studies have focused on enhancing the polyphenols' bioavailability, especially RES and curcumin, by employing nanoformulations, such as lipid-based nanoparticles (liposomes), protein-based nanoformulations, polymers, micelles, and metal nanoparticles for drug delivery (281). For instance, RES and curcumin co-delivery by polymeric micelles to doxorubicin-treated cardiomyocyte cell lines resulted in higher drug solubility and efficacy than non-polymeric forms and single-drug treatment (282). Polymeric micelles used in these studies are biocompatible and FDA-approved. Moreover, polymer-based drug delivery vehicles such as Poly (D,L-lactic-co-glycolic acid) (PLGA) nanoparticles demonstrated superior anti-atherosclerotic effects of curcumin-bioperine treatment compared to non-coated ones. Pillai et al. reported that curcumin-bioperine coated with PLGA nanoparticles significantly reduced ox-LDL levels, downregulating the atherosclerotic plaque-related gene expression *in vitro* (283). The efficacy of PLGA-encapsulated curcumin was also illustrated *in vivo* in later studies, where gold-capped curcumin encapsulated within PLGA demonstrated enhanced solubility, bioavailability, and eventually, improved anti-hypertrophic characteristics in hypertrophic Wistar rat models (284).

Aside from polymeric-based drug delivery, extensive studies were carried out on the efficacy of liposome-targeted therapy (285–287). Liposomes are phospholipid-based nanocarriers designed for both hydrophilic and hydrophobic drug delivery. Liposome preparation progress in recent years has introduced the ligand surface-engineered and long-circulating liposomes, allowing stability and sustained drug release in target tissues and enhanced bioavailability (288). The use of liposomes in CVDs has become a promising candidate for future safe drug delivery without cytotoxicity. RES-contained liposomes were demonstrated to enhance the respiratory capacity of the cardiomyocytes *in vitro* (289). Moreover, the transplantation of mitochondrial-activated cardiac progenitor cells by liposomal RES into the heart of the mouse models with cardiomyopathy resulted in reduced oxidative stress and apoptotic activities of the cardiomyocytes (290).

Considering their minimal side effects and significant therapeutic features, polyphenols have introduced themselves as intriguing options for future HF treatment. However, due to the low bioavailability and stability of polyphenols in the circulating system, limited clinical trials have been conducted evaluating their impact on human HF, and most of the studies are *in vitro* and *in vivo* animal models. However, with recent advances in novel drug delivery systems, there is rising hope for translating nanoparticle delivery of these compounds to the clinical setting soon.

## 9. Conclusions

In conclusion, this study predominantly reviewed the therapeutic effects of polyphenol compounds and their underlying mechanisms in improving pathologic cardiac remodeling, leading to heart failure. Also, this study discusses the most recent advances and challenges in translating the antioxidative, anti-inflammatory, anti-apoptotic, and antifibrotic characteristics of these nutraceuticals into clinical settings. The potential application of nano-drug delivery systems was also investigated regarding the limited solubility, stability, and bioavailability of the polyphenols in humans. Finally, further in-depth surveys are needed to understand better the involved molecular pathways of polyphenols in modulating cardiac remodeling and HF. Also, there is a rising demand for viable strategies to better translate the *in vitro* and animal study results into the clinic.
